# Army Drift

**Published:** 1898-08

**Authors:** 


### ARMY DRIFT.

Now that affairs in connection with our army are being dealt with in the daily press, it is well for the people at large to better know the actual condition of the army veterinarians. While a professional man, he is rarely if ever consulted in matters pertaining to his profession, except in the treatment of sick and disabled horses, and in that he is badly hampered by reason of the inadequate supply of drugs and instruments, which is now better known, since the public have gained information about the lack of needful remedies in dealing with sick soldiers. Even the purchases of these drugs and instruments are through incompetent sources, and are drawn quarterly by troop commanders, usually without any reference to the needs of the veterinarian, thus causing double loss, and unnecessary instruments are purchased, as well as useless ones, and surgical treatment is not what it should be on account of the lack of proper instruments.

Army veterinarians, as a class, keep themselves well posted with the advance of medical science, and when new remedies or a specially pure drug are needed for any unusual case, they can only be had by special requisition on the Quartermaster Department, which, as usual, has to go through the "Circumlocutionary Office," so that on its arrival (provided it is allowed), the patient's bones are quite as likely to be bleaching on the prairie.

In the supply of food-products, especially that of meat and milk, there are none so well qualified for such inspection as this as the veterinarian, yet the supply of meat is received and inspected by young officers who do not pretend to have any knowledge of such matters. Is it not time that this uncalled-for state of affairs should cease?

In these days, demanding proper sanitary conditions in the production of milk and its products, cream and butter, there is no longer any excuse for these to be supplied from cows stabled in unsanitary huts, where their principal sustenance in winter is derived from stable manure and refuse. Men's systems sustained from food-products with no better safeguards around them than these are not fit for hardship and privations in times of war, and we need not wonder at the prevalence of tuberculosis among many of the soldiers under such conditions as these.

Horses condemned for acute and chronic diseases (laminitis, navicular, etc.) are, like all others, sold at auction for a nominal sum, and have to end up their agonizing existence under new and exacting masters, when they should have been humanely destroyed.

The loss by death and condemnation is quoted at about 25 per cent., but this would be very much greater were it not for the aid many times given by the private medicine-case of the veterinarians of the army, who keep in touch with the advancement of veterinary science.

The purchase of cavalry and artillery horses, as well as quartermaster's animals, by incompetent persons and through city contractors is one of the sources of the greatest loss, and impossible to estimate, many being physically unsound and in conformation wholly unsuitable for service, though they have been purchased at the extravagant price of upward of $150 in times of peace.

A recent statement in one of the leading livestock journals of Chicago, showing where fifty horses were passed in sixty minutes, needs no further comment.

Prior to the inauguration of the war just closed there were about twelve thousand horses in the regular cavalry, which at $150 each cost $1,800,000; therefore the losses by condemnation and death of 25 per cent. represent $450,000 annually.

Cavalry horses are condemned by an officer (inspector) who visits posts annually. The animals are submitted by the troop commanders many times without consulting the veterinarian, though neither inspector nor troop commander has any knowledge of matters veterinary. In this way frequently good horses are condemned and poor ones retained. Why not wipe out the entire veterinary force of the army rather than continue it in its present condition.

During the recent war a board of cavalry officers, assisted by the army veterinarian, were sent on purchasing tours through the West, and it is to be noted at this time that in every way the animals purchased in this manner were more serviceable than any furnished by the quartermaster's department, and were in every way superior so far as physical soundness and being well-broken. The price, fixed at $100, as an emergency one, would have been, under ordinary conditions, reduced to half that figure, though at $100 per head $50 each was saved. On the purchase of $600,000 worth of horses there is represented a clear saving of $150,000. Here may be noted one of the reasons why army veterinary legislation has its opponents, because these matters would have a thorough sifting and the Government would be saved thousands of dollars.

The question of forage is an all-important one. Like food-products for the soldiers, it is received and inspected by a young officer acting as quartermaster, without any education or special knowledge in this direction, and the annual loss from this source is very great, and many times even this officer relegates this duty to a subordinate who knows no more and cares less. Often inferior and innutritious in quality, and having undergone changes which make it a dangerous food, yet the veterinarian must deal with the sickness resulting therefrom and suffer any reflection or criticism that may be passed upon him, though powerless to prevent these disastrous results that are sure to follow the purchase of such provender. Twelve pounds of oats and fourteen pounds of hay, with three pounds of bedding, are the daily rations. A saving might be made here under proper veterinary supervision and in times of peace and under special climatic conditions known to the experienced army veterinarian might be made with benefit to the animals and a great saving to the Government. Thirty-six thousand pounds of oats per day, at two cents per pound for one hundred and eighty days, would represent a saving of $129,600. Why does this reckless extravagance go on? This would only represent a reduction in the daily ration of three pounds of oats per day.

We sum up to find the annual loss by condemnation to be $450,000, a saving that could be made annually with advantage to the service—in grain of $129,600; in the purchase of cavalry horses annually (not war times) $150,000; losses incidental to lack of proper drugs, instruments, etc., $100,000, with a total of $829,600. Such are the figures in the regular cavalry branch alone. If to this were added artillery and quartermasters' animals, the sum would be increased enormously.

Let us suppose that the loss annually is one million dollars, and to those who wish to question it, divide it in half, thus saving a half million dollars. Why do not those legislators at Washington, who have been designated as the "watch-dogs" of the treasury, see to the closing up of this great leak and give to the veterinarians of the army the necessary rank and commission that give to them the power to control matters and no longer stand aghast at this extravagance and be cajoled into its continuance by the "dog in the manger" in the army.

Our vanquished enemy, Spain, where it is said that only one out of every five of her people can read and write, does not for a moment tolerate such a condition of affairs as this.

The army is in the hands of Congress, and now that retrenchments will be demanded on all sides in order that our people shall not be continued any longer than necessary under war taxes, as they have been for thirty years, there should be a revolution in the management of our army department, and this pernicious and blighting state of affairs remedied.

"Burnt-leather inhalations" prevail as a sovereign remedy among the farriers, or self-styled "horse-doctors," in the various troops and companies.

The Buffalo *Horse World*, in its issue of August 12th, strongly urges editorially the establishment of a proper veterinary corps for our army, and points out the great unnecessary losses.

Every influence should be exercised in urging favorable consideration by the War Department of the present Army Bill before Congress. It has the favor of Chairman Hawley, of the Senate, and General Hull, of the House, and it received the indorsement of Adjutant General Ruggles before his retirement.

It is rumored that the social aspect of the issue of recognition to army veterinarians stands also in the way, and for this taxes raised from our people to the extent of nearly ten millions of dollars now invested in livestock for our army stands imperilled because of the lack of an efficient army veterinary medical corps.

Our defeated and now third-rate nation, Spain, fully realized the importance of the care of her horses in army service, and no such reports are heard relative to the neglect of her horses and mules, sickness without attention, reckless buying and shipping of such perishable wealth as have been repeated so often here from every shipping-point and camp. Spain's veterinary corps consists of one first-class veterinary inspector (colonel); two second-class veterinary inspectors (lieutenant-colonels); nine veterinary majors; seventy-three veterinary captains; eighty-seven veterinary lieutenants; some two hundred and thirty-five in all.

---

Much comment is indulged in as to the Government spending large sums of public moneys in the preparation of a hog-cholera serum, the formula of which they refuse to disclose. This leans more than a little to the secret-remedy pursuit, and many are asking if this is a legitimate field of our Government.

